# “Conical” Frustum Multi-Beam Phased Arrays for Air Traffic Control Radars

**DOI:** 10.3390/s22197309

**Published:** 2022-09-27

**Authors:** Paolo Rocca, Nicola Anselmi, Mohammad Abdul Hannan, Andrea Massa

**Affiliations:** 1 DICAM—Department of Civil, Environmental, and Mechanical Engineering, ELEDIA Research Center, ELEDIA@UniTN—University of Trento, Via Mesiano 77, 38123 Trento, Italy or; 2 ELEDIA Research Center, ELEDIA@XIDIAN—Xidian University, No. 2 South Tabai Road, Xi’an 710071, China; 3 ELEDIA Research Center, ELEDIA@UESTC—UESTC, School of Electronic Engineering, Chengdu 611731, China; 4 ELEDIA Research Center, ELEDIA@TSINGHUA—Tsinghua University, 30 Shuangqing Rd, Beijing 100084, China

**Keywords:** phased array, conical frustum array, sparse array, multi-beam array, digital beamforming, compressive sensing, radar, air traffic control

## Abstract

The design of conical frustum phased array antennas for air traffic control (ATC) radar systems is addressed. The array architecture, which is controlled by a fully digital beam-forming (DBF) network, is composed by a set of equal vertical modules. Each module consists of a linear sparse array that generates on receive multiple instantaneous beams pointing along different directions in elevation. To reach the best trade-off between the antenna complexity (i.e., minimum number of array elements and/or radio frequency components) and radiation performance (i.e., matching a set of reference patterns), the synthesis problem is formulated in the Compressive Sampling (CS) framework. Then, the positions of the array elements and the complex excitations for generating each single beam are jointly determined through a customized version of the Bayesian CS (BCS) tool. Representative numerical results, concerned with ideal as well as real antenna models, are reported both to validate the proposed design strategy and to assess the effectiveness of the synthesized modular sparse array architecture also in comparison with conventional arrays with uniformly-spaced elements.

## 1. Introduction

Surveillance systems are crucial elements in airports for the correct execution of all terminal functions and procedures such as airplanes take off and landing, air traffic management, and terminal security. Nowadays, most air traffic control (ATC) radars use ground stations, equipped with large reflectors and/or array antennas installed on mechanical rotating pedestals, to yield a $360^{\circ }$ view for tracking the targets all around. Such a technology, in place since the 1970s [1], is currently close to upgrade and novel solutions, based on cylindrical or multi-face planar phased arrays (PAs) [2,3,4,5], are currently investigated. Thanks to the rapid scan, the clutter suppression, the multi-beam generation, and the multi-function capabilities (e.g., joint ATC and weather radar functionalities [6,7,8]), electronically scanning PA-based solutions with DBF seem to be promising candidates for the next generation of ATC radar systems [9,10]. Indeed, compared to an analog beam-forming (ABF), the DBF has the advantage of simultaneously generating multiple beams on receive with arbitrary shapes. Moreover, DBF allows a very accurate pointing over wide bandwidths by avoiding the beam squint effects thanks to the use of true time delays [11].

ATC radars require narrow beams to achieve high resolution and, consequently, they need PAs with large apertures and a huge number of radiating elements as well as analog-digital converters (ADCs). Although the cost, the size, and the power consumption of ADCs are continuously decreasing, the use of PAs with classical/fully-populated (FP) architectures (i.e., array layouts with uniformly-spaced radiating elements, each one equipped with a dedicated ADC) would result too expensive for commercial applications. Therefore, scalable and modular DBF implementations are of great interest for future fully-digital ATC PA-based radars. In this framework, innovative unconventional array architectures have been recently proposed to implement better cost-performance trade-off solutions [12]. For instance, clustered/sub-arrayed PAs have been considered for reducing the number of control points by gathering multiple elements over a single input-output port to minimize the production and maintenance costs [13,14,15,16,17,18,19,20]. The main issue when resorting to clustered arrays is the unavoidable presence of undesired secondary lobes (i.e., the *quantization lobes* [21,22]). To cope with this drawback, irregular arrangements of the array clusters have been exploited and the optimization of the sub-array configurations (i.e., sub-array position/size/shapes and excitations) has been carried out by means of various strategies [13,14,15,16,17,18,19,20].

Alternatively, another effective approach to simplify the PA architecture is array sparsening [23,24,25,26,27,28,29,30,31,32,33]. By jointly optimizing the inter-element distances between the array elements and the corresponding excitation weights, the number of array elements is minimized, while fitting user-defined radiation performance. Deterministic [24,32] and stochastic techniques [23,25] as well as Compressive Sensing (CS) based methods [26,27,28,29,30,31,33,34] have been published in the recent scientific literature to synthesize sparse arrays.

This paper presents a DBF PA antenna for ATC radar systems, which has been preliminary introduced [35], that benefits from both the modularity and the sparseness of the array layout. More specifically, the array is composed by sparse vertical planks (i.e., sub-array modules), to minimize the number of antenna elements and of the corresponding ADCs, positioned side-by-side over a truncated conical surface. The choice of a conical frustum shape is quite natural since the surface is already tilted towards the sky, thus a lower elevation scanning is needed [36,37,38], while the DBF simultaneously generates multiple beams to cover the required elevation range. Thanks to the modular architecture along the vertical direction and the circular symmetry, the array affords a $360^{\circ }$ view along the azimuth plane. Moreover, the processing of the signals collected by a contiguous subset of the array planks guarantees uniform radar performance [39].

To determine the linear sparse arrangement of the elementary radiators of the vertical plank and the corresponding excitation sets, the synthesis problem is cast as an optimization one aimed at simultaneously generating multiple beams, as close as possible to those radiated by an ideal/reference FP faithfully fulfilling the user requirements, while jointly minimizing the number of elements. Towards this end, a sparse-regularization technique based on the Bayesian CS (BCS) technique is here exploited because of its proved efficiency and effectiveness in finding the sparsest linear [26,27], planar [30] and conformal [31] array layouts and the complex beam-forming weights that minimize the pattern matching misfit. However, none of the previous BCS synthesis methods [27,30,31] has dealt with the joint generation of multiple simultaneous radiation patterns from a common sparse array layout. Therefore, this work introduces a novel synthesis strategy based on a Multi-Task (MT) [40] implementation of the BCS where the condition of common positions for the array elements is enforced, while optimizing an independent set of complex (amplitude and phase) excitations for simultaneously generating multiple independent beams. The arising non-convex synthesis problem, not solvable by running a set (i.e., one for each beam) of parallel BCS-based optimizations because of the need to enforce a common array layout, is addressed by minimizing the *a-posteriori* probability for the array to jointly match the target patterns by means of a fast Relevance Vector Machine (RVM) solver [40]. The BCS has demonstrated requiring a computation burden several orders in magnitude lower than global optimization methods as well as the capability of easily including user-defined constraints on the array layout, not admissible with analytic techniques [34].

To the best of the authors knowledge, the main novelties of this work with respect to the state-of-the-art literature include (*i*) the introduction of an innovative modular architecture (i.e., a conformal/conical multi-beam sparse receiving array) for ATC radar systems; (*ii*) the theoretical formulation of the design problem within the CS framework to yield a solution that assures the best compromise/trade-off between the closeness/fulfilment of the user/operative radiation requirements and the reduction of the architectural complexity (i.e., the minimum number of elements/ADCs); (*iii*) the development of a synthesis tool based on a customized implementation of the BCS to jointly synthesize the array layout and the sets of complex excitations for generating the multiple beams.

The rest of the paper is organized as follows. The synthesis of conical frustum fully-digital arrays for ATC radars is mathematically formulated in Section 2, where the proposed MT-BCS based solution method is described, as well. Section 3 presents a set of selected numerical results to validate the synthesis method and to assess the effectiveness of the arising layouts also considering realistic antenna models. Eventually, conclusions follow (Section 4).

## 2. Mathematical Formulation

**Figure 1:**
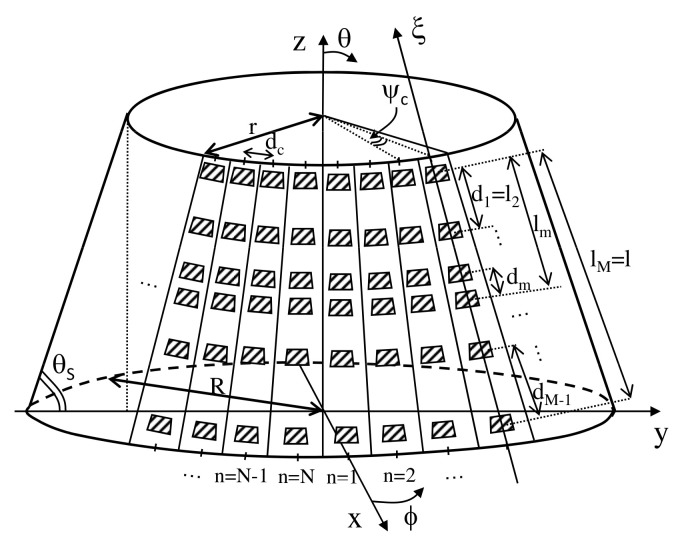
Sketch of the sparse CPA geometry.

**Figure 2:**
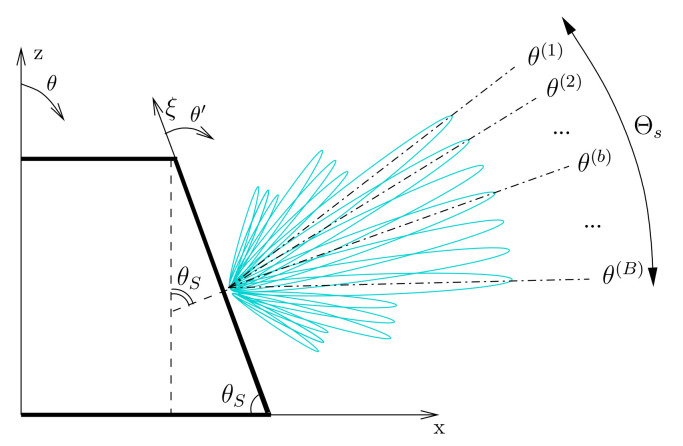
Sketch of the transversal section of the CPA and of the radiation mechanism.

Let us consider a conical-frustum phased array (CPA) of *P* radiating elements located on the surface of a truncated cone with axis along the *z* Cartesian coordinate and circular bases lying on the (*x*,*y*)-plane with radii *R* (major base radius) and *r* (minor base radius), respectively (Figure 1). Such a CPA is composed by *N* vertical planks of *M* elements (P≜M×N) placed side-by-side at a constant distance $d_{c}$ measured along the circular perimeter of the minor base of the cone. To yield a modular CPA architecture, all *N* planks are equal and they consist of a sparse linear distribution of the elementary radiators over a regular lattice, $d_{m}$ (m=1,…,M−1) being the inter-element distance between the *m*th and $\left(m+1\right)$th adjacent elements, while *l* ($l≜\sum_{m=1}^{M-1}d_{m}$) is the length of the plank [i.e., the distance between the first (i.e., m=1) and the last (i.e., m=M) elements along the plank axis]. Thus, $r=\frac{d_{c}N}{2\pi }$ and $R=r+l\cos\theta_{S}$, $\theta_{S}$ being the cone slant angle (Figure 1 and Figure 2), while the coordinates of the position of the *m*th (m=1,…,M) element in the *n*th (n=1,…,N) array-column/plank turn out to be shown in Equation (1)

(1) $$\begin{array}{c} x_{m,n}=\left[R-\left(l-l_{m}\right)\cos\theta_{S}\right]\cos\left[\left(n-1\right)\psi_{c}\right]\\ y_{m,n}=\left[R-\left(l-l_{m}\right)\cos\theta_{S}\right]\sin\left[\left(n-1\right)\psi_{c}\right]\\ z_{m,n}=\left(l-l_{m}\right)\sin\theta_{S} \end{array}$$

where $\psi_{c}$ ($\psi_{c}≜\frac{2\pi }{N}$) is the angular distance between the centers of two planks (Figure 1) and $l_{m}$ (m=1,…,M) is the distance between the first element and the *m*th one, being $l_{1}=0$ and $l_{M}=l$.

**Figure 3:**
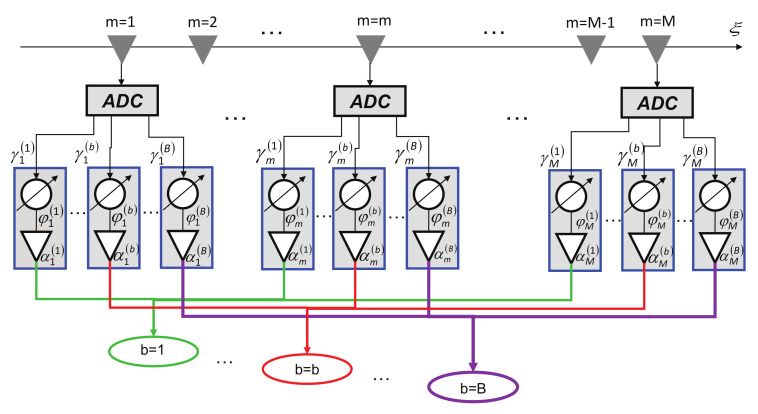
Logical scheme of the DBF network of the single plank that generates *B* independent radiation beams.

The generation of *B* pencil beams (Figure 2) pointing along different elevation directions, {$\left(\theta^{\left(b\right)},\phi^{\left(b\right)}\right)$; b=1,…,B}, is obtained by controlling the amplitude and the phase weights of the CPA excitations by means of the fully DBF architecture sketched in Figure 3. More in detail, the signal received by each antenna is digitized with an ADC and it is weighted by the set of amplitude weights, α (α≜ {$\alpha_{m,n}^{\left(b\right)}$; m=1,…,M; n=1,…,N; b=1,…,B}), and phase delays, φ (φ≜ {$\varphi_{m,n}^{\left(b\right)}$; m=1,…,M; n=1,…,N; b=1,…,B}), that are combined to afford the *B* independent patterns, each *b*th (b=1,…,B) beam pointing towards the user-defined angular direction $\left(\theta^{\left(b\right)},\phi^{\left(b\right)}\right)$. The electromagnetic far-field pattern of the *b*th beam (b=1,…,B) radiated by the CPA is shown in Equation (2)

(2) $$E^{\left(b\right)}\left(\theta ,\phi \right)=\sum_{n=1}^{N}\sum_{m=1}^{M}e_{m,n}\left(\theta ,\phi \right)a_{m,n}\left(\theta ,\phi \right)\gamma_{m.n}^{\left(b\right)}$$

where $e_{m,n}\left(\theta ,\phi \right)$ is the embedded/active*-*element pattern [21,22],

(3) $$\begin{array}{c} a_{m,n}\left(\theta ,\phi \right)=\exp\left\{j\frac{2\pi }{\lambda }\right.\\ \left[x_{m,n}\left(\sin\theta \cos\phi -\sin\theta^{\left(b\right)}\cos\phi^{\left(b\right)}\right)+\right.\\ y_{m,n}\left(\sin\theta \sin\phi -\sin\theta^{\left(b\right)}\sin\phi^{\left(b\right)}\right)+\\ \left.\left.z_{m,n}\left(\cos\theta -\cos\theta^{\left(b\right)}\right)\right]\right\}\;, \end{array}$$

is the steering vector, and $\gamma_{m.n}^{\left(b\right)}=\alpha_{m,n}^{\left(b\right)}e^{j\frac{2\pi }{\lambda }}$ is the complex excitation weight of the $\left(m,n\right)$th (m=1,…,M; n=1,…,N) radiator, λ being the free-space wavelength at the working frequency $f_{0}$. The array factor corresponding to the *b*th beam (b=1,…,B), independent of the type of radiators used in the practical array, is given by $F^{\left(b\right)}\left(\theta ,\phi \right)=\sum_{n=1}^{N}\sum_{m=1}^{M}a_{m,n}$$\left(\theta ,\phi \right)\gamma_{m.n}^{\left(b\right)}$.

Because of the modularity of the CPA at hand and that pointing the mainlobe towards the *b*th (b=1,…,B) direction, $\left(\theta^{\left(b\right)},\phi^{\left(b\right)}\right)$, means analytically adding to the $\left(m,n\right)$th (m=1,…,M; n=1,…,N) digital phase coefficient, $\varphi_{m,n}^{\left(b\right)}$, a shift value, as shown in Equation (4) $\Delta \varphi_{m,n}^{\left(b\right)}$

(4) $$\begin{array}{c} \Delta \varphi_{m,n}^{\left(b\right)}=-\frac{2\pi }{\lambda }\left(x_{m,n}\sin\theta^{\left(b\right)}\cos\phi^{\left(b\right)}+\right.\\ \left.y_{m,n}\sin\theta^{\left(b\right)}\sin\phi^{\left(b\right)}+z_{m,n}\cos\theta^{\left(b\right)}\right)\;, \end{array}$$

the degrees of freedom (DoFs) in designing such a CPA architecture turn out to be only those of a single plank, namely the positions of the *M*-element sparse linear array of the plank and the corresponding amplitude, $\alpha^{\left(b\right)}=\left\{\alpha_{m}^{\left(b\right)};\;m=1,\ldots ,M\right\}$ (b=1,…,B), and phase, $\varphi^{\left(b\right)}=\left\{\varphi_{m}^{\left(b\right)};\;m=1,\ldots ,M\right\}$ (b=1,…,B), excitation coefficients, being $\alpha_{m,n}^{\left(b\right)}=\alpha_{m}^{\left(b\right)}$ and $\varphi_{m,n}^{\left(b\right)}=\varphi_{m}^{\left(b\right)}-\frac{2\pi }{\lambda }\left(x_{m,n}\sin\theta_{S}+z_{m,n}\cos\theta_{S}\right)$ (m=1,…,M; n=1,…,N). The design of the CPA is then cast as the synthesis of the linear sparse array, which composes the vertical plank, that simultaneously generates ***B*** beams. Mathematically, it can be stated as follows:

*Multi-Beam Sparse Array Synthesis Problem* (MBSASP)—Given a set of *B* reference beam patterns, {${\hat{F}}^{\left(b\right)}\left(\theta^{\prime }\right)$; b=1,…,B}, radiated by a reference FP linear array of *I* elements uniformly spaced by *d* and pointing towards *B* directions along the elevation plane (Figure 2), {$\theta^{\left(b\right)}$; b=1,…,B}, determine the corresponding *M*-element (M<I) maximally sparse arrangement and the set of *B* amplitude $\alpha^{\left(b\right)}$ and phase $\varphi^{\left(b\right)}$ excitation coefficients (b=1,…,B), such that *M* is minimum and the multi-beam pattern matching constraint

(5) $$\frac{\sum_{b=1}^{B}\sum_{k=1}^{K}{\left|{\hat{F}}^{\left(b\right)}\left(\theta_{k}^{\prime }\right)-F^{\left(b\right)}\left(\theta_{k}^{\prime };\;\alpha^{\left(b\right)},\;\varphi^{\left(b\right)}\right)\right|}^{2}}{BK}<\epsilon$$

is satisfied, {$\theta_{k}^{\prime }$; k=1,…,K} being the set of *K* sampling directions along the elevation plane, while ϵ is the user-defined parameter controlling the degree of accuracy of the patterns matching.

**Figure 4:**
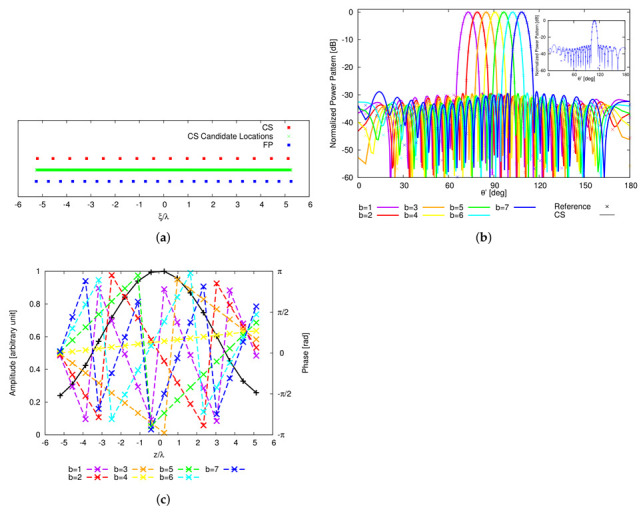
Numerical Assessment (M=16, $d=\frac{\lambda }{2}$, B=7)—Plot of (**a**) the MT-BCS synthesized layout of the plank and of (**b**) the *B* radiated power patterns when the sparse CPA is fed with (**c**) the corresponding sets of complex excitations.

To solve the MBSASP, let us consider a coordinate system with the ξ-axis along the plank column (Figure 1 and Figure 2) so that the corresponding broadside direction, $\theta^{\prime }=90^{\circ }$, coincides with the slant angle $\theta =\theta_{S}$ of the reference coordinate system (Figure 2), being $\theta^{\prime }=\theta +\left(90^{\circ }-\theta_{s}\right)$, and the non-uniform positions of the *M* elements of the plank, {$\xi_{m}$; m=1,…,M}, belong to a set of *Q* (Q≫M) user-defined candidate locations, {$\xi_{q}$; q=1,…,Q}, of a uniform lattice [see for instance Figure 4a). Accordingly, the synthesis of the *B* sets of complex excitation weights $\gamma^{\left(b\right)}$ ≜ {$\gamma_{q}^{\left(b\right)}=\delta_{qm}\alpha_{m}^{\left(b\right)}e^{j\varphi_{m}^{\left(b\right)}}$; q=1,…,Q; m=1,…,M}, $\delta_{qm}$ being the Kronecker function ($\delta_{qm}=1$ if $\xi_{q}=\xi_{m}$ and $\delta_{qm}=0$, otherwise), is mathematically formulated as Equation (6)

(6) $$\gamma_{\ell_{0}}^{\left(b\right)}=\arg\left\{\min_{\gamma^{\left(b\right)}}{∥\gamma^{\left(b\right)}∥}_{\ell_{0}}\right\}\;$$

subject to Equation (7)

(7) $${\hat{F}}^{\left(b\right)}-A\gamma^{\left(b\right)}=\eta^{\left(b\right)}\;\;\;\;b=1,\ldots ,B$$

where A is the steering matrix ($A≜\left\{a_{kq}=e^{j\frac{2\pi }{\lambda }\xi_{q}\cos\theta_{k}^{\prime }};\;\right.$ $\left.k=1,\ldots ,K;\;q=1,\ldots .,Q\right\}$), ${\hat{F}}^{\left(b\right)}$ (${\hat{F}}^{\left(b\right)}≜\left\{{\hat{F}}^{\left(b\right)}\left(\theta_{k}^{\prime }\right);\right.$ $\left.k=1,\ldots ,K;\;q=1,\ldots .,Q\right\}$) is the set of *K* samples of the *b*th (b=1,…,B) reference pattern, while $\eta^{\left(b\right)}=\left\{\eta_{k}^{\left(b\right)};\;k=1,\ldots ,K\right\}$ is the *b*th (b=1,…,B) noise vector whose entries are zero-mean Gaussian complex error values with variance σ proportional to ϵ.

The MT-BCS strategy [27] is then adopted and customized to deal with the ill-posed/ill-conditioned problem in Equation (6) by statistically correlating the *B* sets of array excitations. This implies to enforce the linear array having non-null excitations at the same positions of the *Q*-locations uniform lattice so that the sparsest array layout (i.e., the minimum value of *M*) is retrieved. More specifically, each *b*th (b=1,…,B) constraint in Equation (7) is coded into 2 real-valued tasks as shown in Equation (8) (The problem constraints are transformed in a real-valued form to enable the use of *CS*-based state-of-the-art algorithms [34].)

(8) $$\begin{array}{c} \left\{\begin{array}{c} {\tilde{F}}_{R}^{\left(b\right)}-\;\tilde{A}\gamma_{R}^{\left(b\right)}={\tilde{\eta }}_{R}^{\left(b\right)}\\ {\tilde{F}}_{I}^{\left(b\right)}-\;\tilde{A}\gamma_{I}^{\left(b\right)}={\tilde{\eta }}_{I}^{\left(b\right)} \end{array}\right. \end{array}$$

where $\gamma_{R}^{\left(b\right)}≜R\left\{\gamma^{\left(b\right)}\right\}$ and $\gamma_{I}^{\left(b\right)}≜I\left\{\gamma^{\left(b\right)}\right\}$, $R\left\{\cdot \right\}$ and $I\left\{\cdot \right\}$ being the real part and the imaginary one, respectively, ${\tilde{F}}_{R}^{\left(b\right)}≜\left[R\left\{{\hat{F}}_{R}^{\left(b\right)}\right\},\;I\left\{{\hat{F}}_{R}^{\left(b\right)}\right\}\right]$ and ${\tilde{F}}_{I}^{\left(b\right)}≜\left[R\left\{{\hat{F}}_{I}^{\left(b\right)}\right\},\;I\left\{{\hat{F}}_{I}^{\left(b\right)}\right\}\right]$ are two real-valued vectors such that ${\tilde{F}}_{R}^{\left(b\right)}+{\tilde{F}}_{I}^{\left(b\right)}=\left[R\left\{{\hat{F}}^{\left(b\right)}\right\},\;I\left\{{\hat{F}}^{\left(b\right)}\right\}\right]$. Moreover, ${\tilde{\eta }}_{R}^{\left(b\right)}$ and ${\tilde{\eta }}_{I}^{\left(b\right)}$ are also real-valued vectors such that ${\tilde{\eta }}_{R}^{\left(b\right)}+{\tilde{\eta }}_{I}^{\left(b\right)}=\left[R\left\{\eta^{\left(b\right)}\right\},\;I\left\{\eta^{\left(b\right)}\right\}\right]$, and $\tilde{A}≜\left[R\left\{A\right\},\;I\left\{A\right\}\right]$ is a 2K×Q real-valued matrix.

The sparsest weights vectors $\gamma_{R}^{\left(b\right)}$ and $\gamma_{I}^{\left(b\right)}$ (b=1,…,B) are then derived by maximizing, component-by-component, the *a-posteriori* probability of having the $\gamma^{\left(b\right)}$ coefficients in correspondence with the set of reference pattern samples ${\hat{F}}^{\left(b\right)}$

(9) $$\begin{array}{c} \left\{\begin{array}{c} \gamma_{R}^{\left(b\right)}=\arg\left[\max_{{\tilde{\gamma }}_{R}^{\left(b\right)}}P\left({\tilde{\gamma }}_{R}^{\left(b\right)}|{\tilde{F}}_{R}^{\left(b\right)}\right)\right]\\ \gamma_{I}^{\left(b\right)}=\arg\left[\max_{{\tilde{\gamma }}_{I}^{\left(b\right)}}P\left({\tilde{\gamma }}_{I}^{\left(b\right)}|{\tilde{F}}_{I}^{\left(b\right)}\right)\right] \end{array}\right.. \end{array}$$

It turns out to Equation (10) [27]

(10) $$\left\{\begin{array}{c} \gamma_{R}^{\left(b\right)}={\left[\mathrm{diag}\left(\tilde{a}\right)+{\tilde{A}}^{T}\tilde{A}\right]}^{-1}{\tilde{A}}^{T}{\tilde{F}}_{R}^{\left(b\right)}\\ \gamma_{I}^{\left(b\right)}={\left[\mathrm{diag}\left(\tilde{a}\right)+{\tilde{A}}^{T}\tilde{A}\right]}^{-1}{\tilde{A}}^{T}{\tilde{F}}_{I}^{\left(b\right)} \end{array}\right.$$

where $\tilde{a}$ is the shared hyper-parameter vector determined with the RVM solver [40].

Finally, the *b*th (b=1,…,B) set of complex excitations affording the desired *b*th beam is obtained as shown in Equation (11)

(11) $$\gamma^{\left(b\right)}=\gamma_{R}^{\left(b\right)}+j\gamma_{I}^{\left(b\right)}.$$

Unlike phase-only multiple beam synthesis approaches, the possibility to exploit both the excitation amplitudes and phases allows more effective pattern matching, and consequently pattern shaping, performance.

## 3. Numerical Validation

This section has a twofold objective. On the one hand, to assess the effectiveness of a sparse CPA architecture for ATC applications, on the other, the validation of the proposed BCS-based multi-beam synthesis method.

**Figure 5:**
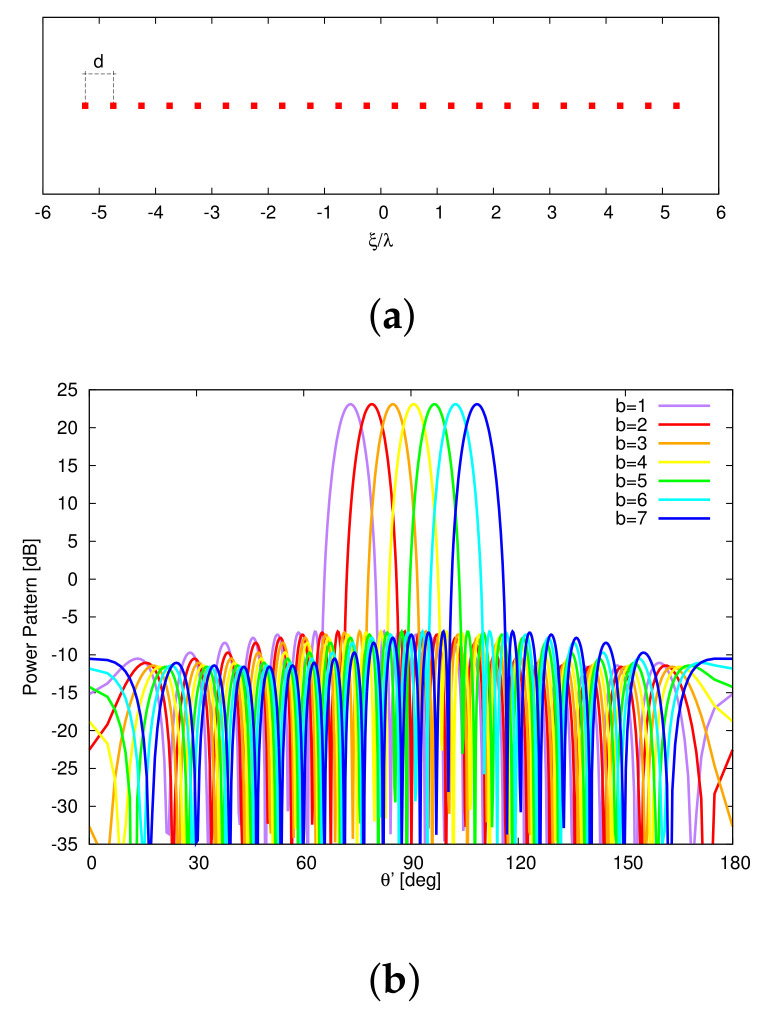
Numerical Assessment (I=22, $d=\frac{\lambda }{2}$, B=7)—Plot of (**a**) the positions of the elements of the FP reference linear array and of (**b**) the *B* radiated (reference) power patterns.

In the benchmark scenario, the CPA has been required to generate B=7 beams pointing at different elevation angles such that each beam intersects at −3 dB the left and the right neighboring beams (Figure 5b) to ensure a −3 dB power coverage within an angular range of $\Theta_{s}=40^{\circ }$ (Figure 2). To span the elevation range $\theta \in \left[50^{\circ }:90^{\circ }\right]$, the slant angle has been set to $\theta_{S}=70^{\circ }$ [36,38], the horizon being at $\theta =90^{\circ }$. Therefore, the *B* steering angles in the plank coordinate system, {$\theta^{\left(b\right)}$; b=1,…,B}, have been set as in Table 1, where the corresponding $-3\;{\mathrm{dB}}_{\mathrm{left}}$ and $-3\;{\mathrm{dB}}_{\mathrm{right}}$ power pattern intersection points at −3 dB and the half power beamwidth (HPBW) are reported, as well.

**Figure 6:**
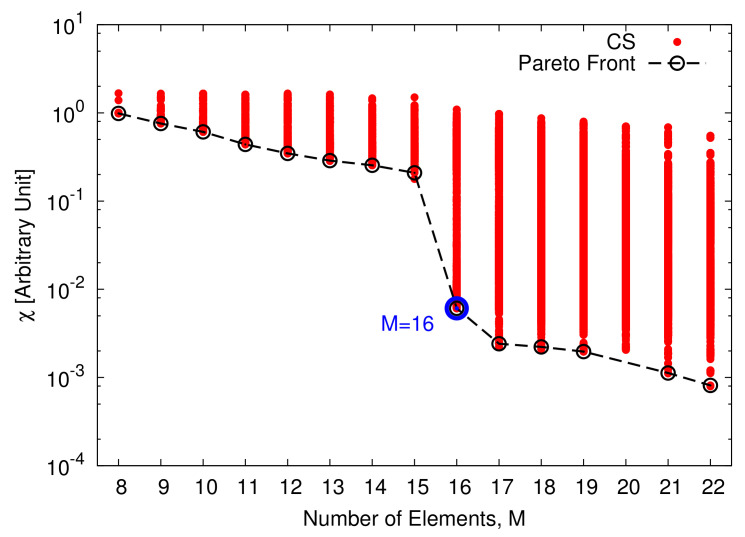
Numerical Assessment (I=22, $d=\frac{\lambda }{2}$, B=7)—Representative points in the (*M*,χ)-plane of the CS-synthesized plank layouts and the corresponding Pareto front.

As a starting point for the MT-BCS synthesis of the single plank, ideal radiators (i.e., $e_{m.n}\left(\theta ,\phi \right)=1$, m=1,…,M, n=1,…,N, 1 being the element pattern of the isotropic antenna) are taken into account. Moreover, the linear configuration of I=22 uniformly-spaced ($d=\frac{\lambda }{2}$ ⇒l=10.5λ) elements in Figure 5a has been used as reference, while the power patterns (Figure 5b) radiated by the corresponding (reference) FP-CPA, composed by N=204 columns spaced by $d_{c}=\frac{\lambda }{2}$ for a total of P=N×I=4488 elements, have been synthesized by tapering the array excitations with a Taylor distribution [41] having SLL=−30 dB and $\bar{n}=6$. These choices resulted in a cone geometry with minor (major) radius equal to r≃16.23λ (R≃19.83λ). Successively, multiple runs of the MT-BCS code have been performed by varying the lattice (i.e., the number of *Q* partitions of *l*: $20\leq \frac{Q}{I}\leq 40$) and the reference pattern sampling (i.e., *K*: $1\leq \frac{K}{I}\leq 3$) as well as the MT-BCS control parameters according to the guidelines in [27,28,29,30,31] (i.e., $\sigma \in \left[10^{-5},10^{-2}\right]$, $\beta_{1}\in \left[10^{-1},10^{4}\right]$, and $\beta_{2}\in \left[5\times 10^{-1},5\times 10^{2}\right]$, $\beta_{1}$ and $\beta_{2}$ being the MT hyper-priors [30]) to explore the achievable trade-offs between pattern matching performance and array sparseness. The degree of optimality of the synthesized layouts has been quantified with the multi-beam power pattern matching error, χ, defined as $\chi ≜\frac{1}{B}\sum_{b=1}^{B}\chi^{\left(b\right)}$ being

(12) $$\chi^{\left(b\right)}≜\frac{\int_{0}^{\pi }\left|{\left|{\hat{F}}^{\left(b\right)}\left(\theta^{\prime }\right)\right|}^{2}-{\left|F^{\left(b\right)}\left(\theta^{\prime }\right)\right|}^{2}\right|d\theta^{\prime }}{\int_{0}^{\pi }{\left|{\hat{F}}^{\left(b\right)}\left(\theta^{\prime }\right)\right|}^{2}d\theta^{\prime }}$$

the single *b*th (b=1,…,B) beam error. The arising Pareto front of the solutions in the M−χ plane is shown in Figure 6. As it can be observed, the error drops down the value of $\chi =6.1\times 10^{-3}$ with at least M=16 elements (Q=700, K=44, $\sigma =10^{-5}$, $\beta_{1}=10^{-1}$, and $\beta_{2}=5\times 10^{-1}$). In this latter case, the array layout is characterized by an aperture of length ${\left.l\right\rfloor }_{M=16}=10.33\;\lambda$ and a maximum (minimum) inter-element spacing equal to $d_{m}^{\max}=0.706\lambda$ ($d_{m}^{\min}=0.646\lambda$) (Figure 4a). The shapes of the B=7 radiated beam patterns are compared to the reference ones in Figure 4b to give an insight on the pattern matching accuracy. For completeness, the *B* sets of the optimized complex excitations, {$\gamma^{\left(b\right)}$; b=1,…,B}, are shown in Figure 4c, while the values of the matching error for each *b*th (b=1,…,B) beam, $\chi^{\left(b\right)}$, are given in Table 2 together with the main pattern descriptors [i.e., the side lobe level (SLL), the peak directivity (D), and the HPBW]. It is worth noticing that the MT-BCS design faithfully matches the whole set of reference patterns (e.g., the maximum degradations of the SLL, of D, and of HPBW being 1.21 dB, 0.04 dBi, and $0.05^{\circ }$, respectively) despite a reduction of 27.3 % of the elements of the FP array. For illustrative purposes, the inset of Figure 4b shows the case with the worst matching error in Table 2 (i.e., b=B=7).

**Figure 7:**
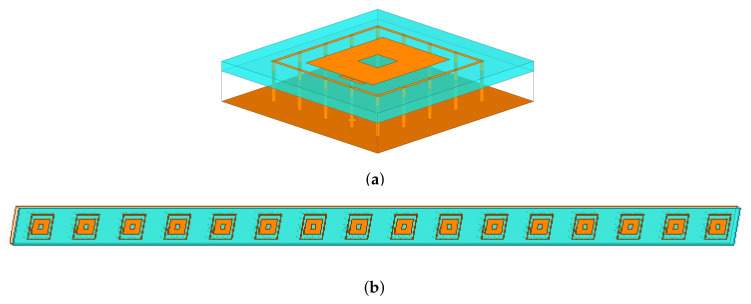
Numerical Assessment (M=16, $d=\frac{\lambda }{2}$, B=7)—Plot of (**a**) the model of the elementary radiator and of (**b**) 16-element sparse linear plank.

**Figure 8:**
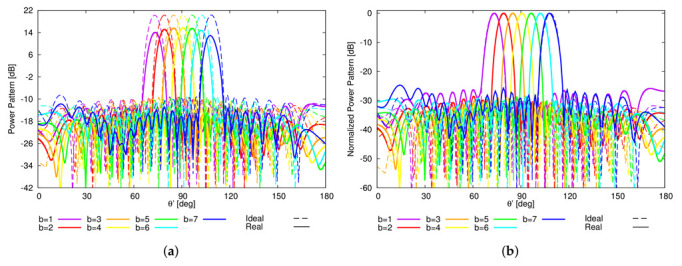
Numerical Assessment (M=16, $d=\frac{\lambda }{2}$, B=7)—Plot of the (**a**) actual and (**b**) normalized *B* radiated power patterns for the ideal and real 16-element sparse linear plank.

In order to assess the reliability of the proposed sparse plank architecture and its robustness against the non-idealities of real arrays, a square-ring microstrip antenna (Figure 7a), suitable for wide angle scanning [42] and resonating in the L-band, has been chosen as elementary radiator of the array. To include the mutual coupling effects of the real array structure, the whole plank model shown in Figure 7b has been simulated at $f_{0}=1.282$ GHz using the finite-element full-wave solver of Ansys HFSS [43]. The comparison of the *B* power patterns radiated by the ideal and real plank model is shown in Figure 8. As it can be observed, apart from the different power level (Figure 8a), the real and the ideal normalized curves overlap in the main beam region, while some differences appear in the far sidelobe region (Figure 8b).

**Figure 9:**
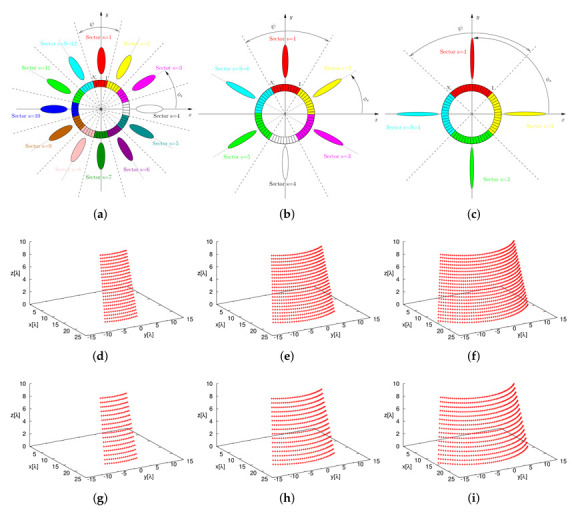
Numerical Assessment (M=16, $d=\frac{\lambda }{2}$, B=7)—CPA configurations when partitioning the lateral surface of the cone in vertical sectors with (**a**,**d**,**g**) $\psi =30^{\circ }$ (→S=12 and $N_{c}=17$), (**b**,**e**,**h**) $\psi =60^{\circ }$ (→S=6 and $N_{c}=34$), and (**c**,**f**,**i**) $\psi =90^{\circ }$ (→S=4 and $N_{c}=51$) widths by using (**d**–**f**) the FP and (**g**–**i**) the sparse planks.

**Figure 10:**
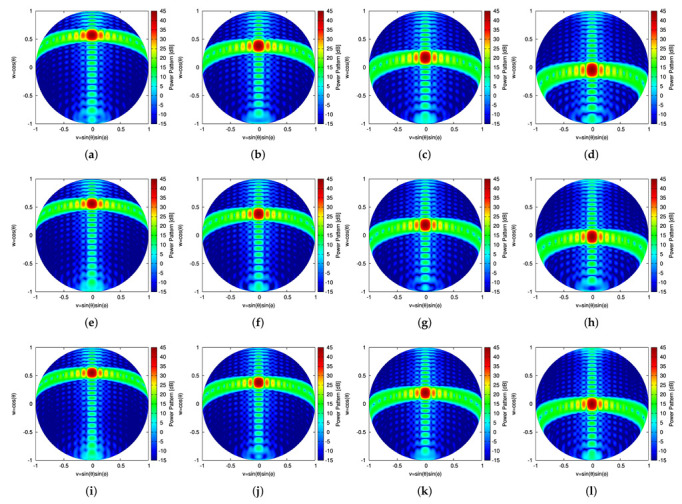
Numerical Assessment (Sparse CPA, M=16, $d=\frac{\lambda }{2}$, B=7, $\psi =30^{\circ }$)—Plot of the power pattern in the (*v*,*w*)-plane of the (**a**,**e**,**i**) b=1, (**b**,**f**,**j**) b=3, (**c**,**g**,**k**) b=5, and (**d**,**h**,**l**) b=7 beams at (**a**–**d**) $f_{min}=1.215$ GHz, (**e**–**h**) $f_{0}=1.282$ GHz, and (**i**–**l**) $f_{max}=1.350$ GHz.

**Figure 11:**
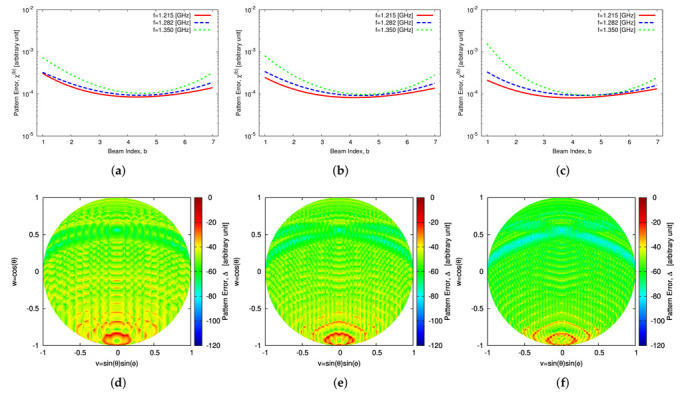
Numerical Assessment (Sparse CPA, M=16, $d=\frac{\lambda }{2}$, B=7)—Plot of (**a**,**c**,**e**) the pattern matching error, $\chi^{\left(b\right)}$, versus the beam index, *b* (b=1,…,B), and of (**b**,**d**,**f**) the local mismatch index, Δ, in the (*v*,*w*)-plane for the b=1 beam at $f_{max}=1.350$ GHz in correspondence with the CPA configurations with vertical sector width equal to (**a**,**b**) $\psi =30^{\circ }$, (**c**,**d**) $\psi =60^{\circ }$, and (**e**,**f**) $\psi =90^{\circ }$.

Once the sparse plank has been synthesized, the CPA has been assembled by subdividing the lateral surface of the cone into *S* vertical sectors, each composed by $N_{c}$ contiguous planks. The $N_{c}$ planks, belonging to the *s*th (s=1,…,S) vertical sector, are responsible of generating *B* beams pointing towards the desired *B* directions along the elevation plane, {$\theta^{\left(b\right)}$; b=1,…,B}, while having the same azimuth angle [i.e., $\phi^{\left(b\right)}=\phi_{s}$ (b=1,…,B)—Figure 9a–c).To assess the performance of the 3D conical-frustum array in focusing the beam along elevation and azimuth, different CPA configurations have been taken into account by setting the angular width of the vertical sector to $\psi =30^{\circ }$ (Figure 9a), $\psi =60^{\circ }$ (Figure 9b), and $\psi =90^{\circ }$ (Figure 9c), which means an architecture of S=12 sectors with $N_{c}=17$ planks (Figure 9a,g), S=6 sectors with $N_{c}=34$ planks (Figure 9b,h), and S=4 sectors with $N_{c}=51$ planks (Figure 9c,i), respectively. Figure 10 shows in a color-map representation the power patterns in the $\left(v,w\right)$-plane (v≜sinθsinϕ, w≜cosθ, being $\theta \in [0^{\circ }:180^{\circ }]$ and $\phi \in [-90^{\circ }:90^{\circ }]$) of a subset (i.e., $b=\left\{1,\;3,\;5,\;7\right\}$) of the B=7 beams radiated by the $\psi =30^{\circ }$ sector CPA. The plots refer to three different frequencies within the L-Band, which is a typical frequency range reserved for aeronautical radio-navigation/radio-localization and, in particular, for Primary Surveillance Radar (PRS) applications [1]. More specifically, the frequencies $f_{\min}=1.215$ GHz (Figure 10a–d), $f_{0}=1.282$ GHz (Figure 10e–h), and $f_{\max}=1.350$ GHz (Figure 10i–l) have been analyzed and the performance of the sparse CPAs (Figure 9g–i) with respect to the reference FP ones (Figure 9d–f) have been evaluated still with the pattern matching metric in Equation (12), but now considering the two angular variables $\left(v,w\right)$ (i.e., $\chi^{\left(b\right)}≜\frac{\int_{v^{2}+w^{2}\leq 1}\left|{\left|{\hat{F}}^{\left(b\right)}\left(v,w\right)\right|}^{2}-{\left|F^{\left(b\right)}\left(v,w\right)\right|}^{2}\right|dvdw}{\int_{v^{2}+w^{2}\leq 1}{\left|{\hat{F}}^{\left(b\right)}\left(v,w\right)\right|}^{2}dvdw}$). The behavior of $\chi^{\left(b\right)}$ versus the beam number (b=1,…,B) at the selected frequencies can be inferred by the plots in first line of Figure 11, which refer to the $\psi =30^{\circ }$ (Figure 11a), the $\psi =60^{\circ }$ (Figure 11c), the $\psi =90^{\circ }$ (Figure 11e) sectorized CPA, respectively. Generally, the error values are in the order of $\chi^{\left(b\right)}\approx 10^{-4}$ and slightly increase ($\chi^{\left(b\right)}\approx 10^{-3}$) only for the border beams (i.e., b=1 and b=B) at the higher frequency (i.e., $f_{\max}=1.350$ GHz). To give the interested readers some insights on the distribution of the error done in approximating the reference pattern within the (*v*,*w*)-plane, a local mismatch index, Δ, has been defined as $\Delta ≜\left|{\left|{\hat{F}}^{\left(b\right)}\left(v,w\right)\right|}^{2}-{\left|F^{\left(b\right)}\left(v,w\right)\right|}^{2}\right|/{\left|{\hat{F}}^{\left(b\right)}\left(v,w\right)\right|}^{2}$ and it has been computed for the worst cases having the greater values of $\chi^{\left(b\right)}$ (i.e., b=1 @ $f_{\max}=1.350$ GHz) (Figure 11—second line]. As it can be inferred, the most significant deviations from the reference pattern turn out to be close to the $\left(\theta ,\phi \right)=\left(180^{\circ },\;0^{\circ }\right)$ [→ $\left(v,w\right)=\left(0,\;-1\right)$] angular direction, that is, far away from the main-beam in the low sidelobe region (Figure 10i).

**Figure 12:**
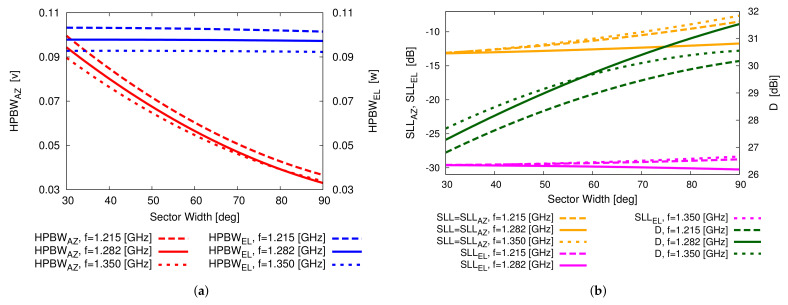
Numerical Assessment (Sparse CPA, M=16, $d=\frac{\lambda }{2}$, b=4, B=7)—Behaviour of (**a**) ${\mathrm{HPBW}}_{\mathrm{AZ}}$ and ${\mathrm{HPBW}}_{\mathrm{EL}}$ and of (**b**) SLL and D versus the vertical sector width, ψ.

**Figure 13:**
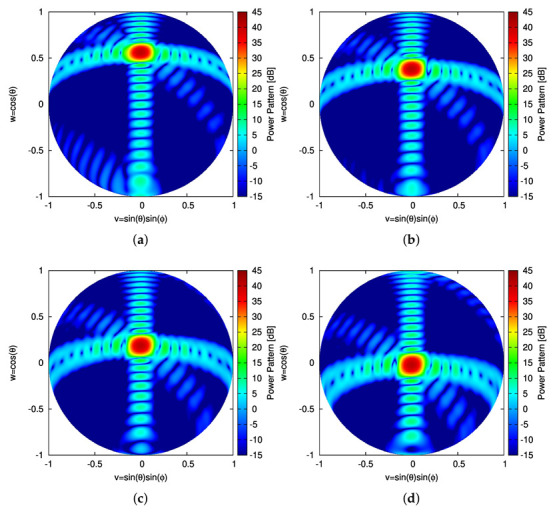
Numerical Assessment (Sparse CPA, M=16, $d=\frac{\lambda }{2}$, B=7, $f_{0}=1.282$ GHz, $\psi =30^{\circ }$)—Plot of the power pattern in the (*v*,*w*)-plane of the (**a**) b=1, (**b**) b=3, (**c**) b=5, and (**d**) b=7 beams when applying a Taylor tapering with SLL=−30 dB and $\bar{n}=4$ to the excitations of the $N_{c}=17$ planks in the azimuth plane.

Next, the main pattern descriptors (i.e., the HPBW along the azimuth (${\mathrm{HPBW}}_{\mathrm{AZ}}$) and elevation (${\mathrm{HPBW}}_{\mathrm{EL}}$), the SLL, the SLL in the elevation plane (${\mathrm{SLL}}_{\mathrm{EL}}≜\mathrm{SLL}\rfloor_{v=0}$), and the peak directivity D) of the different sparse CPA configurations have been analyzed. As a representative example of the whole set of results, the discussion will be focused on the central beam (i.e., b=4). Figure 12a shows the behaviors of ${\mathrm{HPBW}}_{\mathrm{AZ}}$ and ${\mathrm{HPBW}}_{\mathrm{EL}}$ versus the sector width ψ of the CPA architecture. As expected, there is an unavoidably beam broadening effect when increasing the operation frequency and the ${\mathrm{HPBW}}_{\mathrm{AZ}}$ reduces of almost one third widening the angular width of the vertical sector from $\psi =30^{\circ }$ up to $\psi =90^{\circ }$. Concerning the values of SLL and D, which are reported in Figure 12b, it turns out that D increases with the sector width due to the larger size of the aperture that radiates the beam, but the same holds true for the SLL due to the high sidelobes in the azimuth plane since the CPA sector behaves as a uniform array along such a plane (i.e., all planks as well as the element excitations are equal and without tapering along the azimuth plane). However, the azimuth-plane sidelobes can be easily lowered by exploiting, for instance, the pattern multiplication strategy [21,22]. Accordingly, a Taylor taper [41] with SLL=−30 dB and $\bar{n}=4$ has been applied to the amplitudes of the $N_{c}=17$ planks of the sparse CPA assembled with $\psi =30^{\circ }$ vertical sectors. As expected, the plots of the power patterns radiated at the central frequency $f_{0}$ for the $b=\left\{1,\;3,\;5,\;7\right\}$ beams in Figure 13 do not present the high sidelobes along the azimuth plane of the corresponding ones in Figure 10e–h. Indeed, the SLL now turns out to be SLL≤−26.78 dB, which is a value very close to the reference Taylor one.

**Figure 14:**
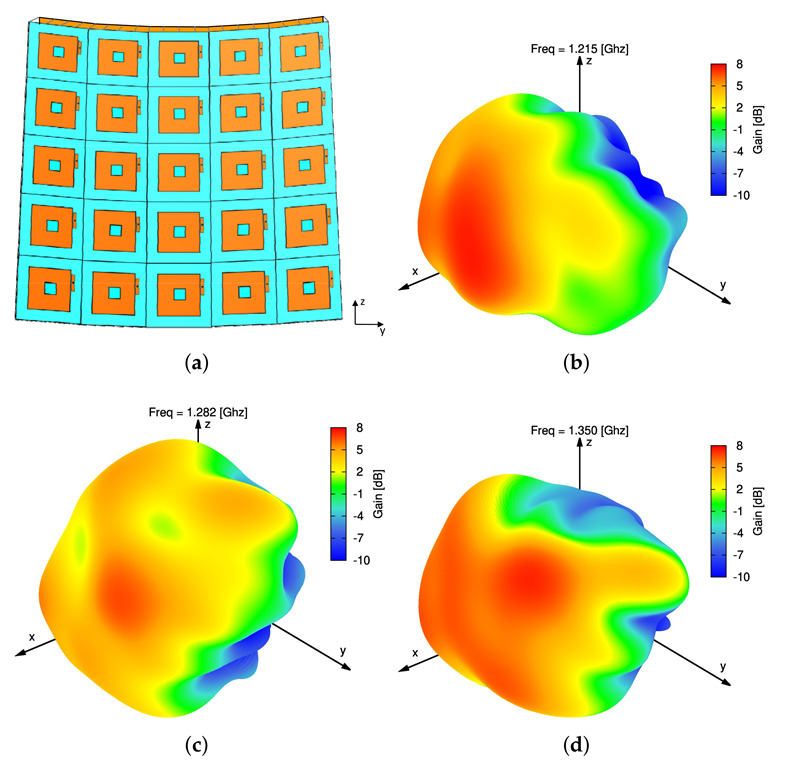
Numerical Assessment—Sketch of (**a**) the 5×5 conformal neighborhood antenna model used to compute (**b**,**c**,**d**) the embedded element pattern at (**b**) $f_{min}=1.215$ GHz, (**c**) $f_{0}=1.282$ GHz, and (**d**) $f_{max}=1.350$ GHz.

**Figure 15:**
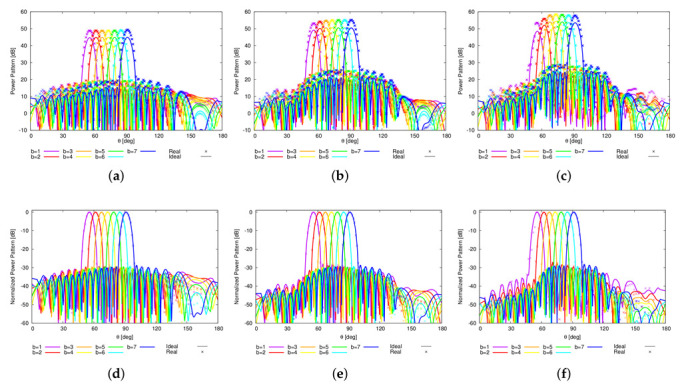
Numerical Assessment (Sparse CPA, M=16, $d=\frac{\lambda }{2}$, B=7, $f_{0}=1.282$ GHz)—Plot along the $\phi =0.0^{\circ }$ cut of the (**a**–**c**) actual and (**d**–**f**) normalized power patterns radiated by the CPA configurations with vertical sector width equal to (**a**,**d**) $\psi =30^{\circ }$, (**b**,**e**) $\psi =60^{\circ }$, and (**c**,**f**) $\psi =90^{\circ }$.

Finally, the behavior of the real model of the proposed sparse CPA modular architecture has been assessed. Towards this end and in order to enable the HFSS full-wave simulation, the far-field patterns of the *B* beams have been computed as $E^{\left(b\right)}\left(\theta ,\phi \right)≃e\left(\theta ,\phi \right)F^{\left(b\right)}\left(\theta ,\phi \right)$ (b=1,…,B), where the embedded element pattern $e\left(\theta ,\phi \right)$, assumed equal for all antennas (i.e., $e_{m,n}\left(\theta ,\phi \right)=e\left(\theta ,\phi \right)$, m=1,…,M, n=1,…,N), has been set to the one of the central element of a neighborhood of 5×5 identical square-ring microstrip antennas (Figure 7a) conformal to the CPA support (Figure 14a). For illustrative purposes, the 3D plots of the embedded pattern at the frequencies of interest are reported: $f_{\min}=1.215$ GHz (Figure 14b), $f_{0}=1.282$ GHz (Figure 14c), and $f_{\max}=1.350$ GHz (Figure 14d). To analyze the radiation performance, Figure 15 compares, along the elevation plane, the power patterns radiated at $f_{0}=1.282$ GHz by the real and the ideal (i.e., $e\left(\theta ,\phi \right)=1$) sparse CPAs in the $\psi =30^{\circ }$ (Figure 15a,d), $\psi =60^{\circ }$ (Figure 15b,e), and $\psi =90^{\circ }$ (Figure 15c,f) configurations. Also in this case, the shapes of the real and the ideal curves show only negligible deviations in the far sidelobe region while the mainlobes are substantially identical (Figure 15d–f).

## 4. Conclusions

The design of a sparse CPA, which generates multiple beams pointing along different elevation directions, to be used as receiver for next-generation ATC radar systems has been carried out. Thanks to a modular structure composed by vertical modules/planks consisting of a sparsely linearly-arranged set of radiating elements, the array, equipped with a fully DBF network to simultaneously generate multiple beams on receive, has been conceived to minimize the architecture complexity and the number of radiating elements as well as ADCs. The CPA synthesis has been carried out by means of a customized implementation of the MT-BCS-based method and it is aimed at jointly optimizing the positions of the plank radiators and the set of complex excitations for generating the multiple beams.

From the numerical assessment with ideal as well as real antenna models, the following main outcomes can be drawn:

- a sparse CPA with 27.3% less elements than the reference FPA, while guaranteeing the same radiation performance, has been synthesized thanks to the proposed CS-based method;
- the effectiveness of the MT-BCS synthesis has been proved also in solving array design problems with multiple concurrent tasks such as the one here addressed and concerned with the simultaneous generation of multiple beams with the same sparse physical architecture;
- the modular structure of the CPA along the elevation plane allows the designer to choose the best trade-off in terms of resolution, radar range, and tracking directions subject to the requirements on the ATC radar at hand.

Future research activities, beyond the scope of this paper, will deal with innovative unconventional CPA architectures that exploit sparsity on both elevation and azimuth to synthesize optimal and dedicated array configurations for the different sector widths since the sparse linear array obtained by means of the proposed method may be not optimal for every array sector. Moreover, other sizes/shapes of the planks to further address the easy-manufacturing and cost-reduction issues of future-generation multi-function radar systems will be investigated. Of course, the extension of current and advanced CPA geometries to other frequency bands will be object of more application-oriented research tracks. 

## Figures and Tables

**Table 1:** Numerical Assessment (FP-CPA, I=22, $d=\frac{\lambda }{2}$, B=7)—Steering angles and HPBW values with respect to the $\theta^{\prime }$ angle.

*b*	$\theta^{\left(b\right)}$	$-3\;{\mathrm{dB}}_{\mathrm{left}}$	$-3\;{\mathrm{dB}}_{\mathrm{right}}$	HPBW
1	$73.07^{\circ }$	$70.00^{\circ }$	$76.09^{\circ }$	$6.08^{\circ }$
2	$79.06^{\circ }$	$76.09^{\circ }$	$82.00^{\circ }$	$5.92^{\circ }$
3	$84.93^{\circ }$	$82.00^{\circ }$	$87.85^{\circ }$	$5.83^{\circ }$
4	$90.75^{\circ }$	$87.85^{\circ }$	$93.65^{\circ }$	$5.81^{\circ }$
5	$96.57^{\circ }$	$93.65^{\circ }$	$99.50^{\circ }$	$5.85^{\circ }$
6	$102.46^{\circ }$	$99.50^{\circ }$	$105.45^{\circ }$	$5.95^{\circ }$
7	$108.47^{\circ }$	$105.45^{\circ }$	$111.55^{\circ }$	$6.13^{\circ }$

**Table 2:** Numerical Assessment (I=22, M=16, $d=\frac{\lambda }{2}$, B=7)—Pattern indexes and pattern matching errors.

*b*	SLL [dB]	D [dBi]	HPBW [${\;}^{\circ }$]	$\chi^{\left(b\right)}$
Reference				
	−30.00	12.77	6.08	
CS				
1	−29.39	12.73	6.12	$7.90\times 10^{-3}$
2	−29.51	12.74	5.96	$5.71\times 10^{-3}$
3	−29.60	12.75	5.87	$4.75\times 10^{-3}$
4	−29.61	12.75	5.85	$4.61\times 10^{-3}$
5	−29.53	12.75	5.87	$5.01\times 10^{-3}$
6	−29.36	12.74	5.99	$5.97\times 10^{-3}$
7	−28.79	12.77	6.13	$8.58\times 10^{-3}$

## Data Availability

Not applicable.

## Nomenclature

The following abbreviations are used in this manuscript:

ABF	Analog Beam-Forming
ADC	Analog-Digital Converter
ATC	Air Traffic Control
AZ	Azimuth
BCS	Bayesian Compressive Sampling/Sensing
CPA	Conical-Frustrum Phased Array
CS	Compressive Sampling/Sensing
D	Directivity
DBF	Digital Beam-Forming
DoF	Degree of Freedom
EL	Elevation
FP	Fully-Populated
HPBW	Half Power Beam-Width
MT	Multi-Task
MBSASP	Multi-Beam Sparse Array Synthesis Problem
PA	Phased Array
PRS	Primary Surveillance Radar
RVM	Relevance Vector Machine
SLL	Side Lobe Level
